# RNA recovery from specimens of duct-washing cytology performed contemporaneously with mammary ductoscopy

**DOI:** 10.1186/s13104-022-05928-1

**Published:** 2022-02-10

**Authors:** Tomoo Jikuzono, Eriko Manabe, Shoko Kure, Haruki Akasu, Tomoko Ishikawa, Yoko Fujiwara, Masujiro Makita, Osamu Ishibashi

**Affiliations:** 1 Department of Endocrine Surgery, Nippon Medical School, 1-1-5 Sendagi, Bunkyo-ku, Tokyo, 113-8602 Japan; 2 Department of Breast Surgery, Nippon Medical School Musashi Kosugi Hospital, 1-396 Kosugi-cho, Nakahara-ku, Kawasaki, 211-8533 Japan; 3 Department of Integrated Diagnostic Pathology, Nippon Medical School, 1-1-5 Sendagi, Bunkyo-ku, Tokyo, 113-8602 Japan; 4 Department of Endocrine Surgery, Nippon Medical School Musashi Kosugi Hospital, 1-396 Kosugi-cho, Nakahara-ku, Kawasaki, 211-8533 Japan; 5 Department of Human Nutrition, Seitoku University, 550 Iwase, Matsudo, Chiba 271-8555 Japan; 6 Institute for Human Life Innovation, Ochanomizu University, 2-1-1 Otsuka, Bunkyo-ku, Tokyo, 112-8610 Japan; 7 Department of Applied Life Sciences, Graduate School of Life & Environmental Sciences, Laboratory of Biological Macromolecules, Osaka Prefecture University, 1-1 Gakuen-cho, Sakai, Osaka 599-8531 Japan

**Keywords:** Mammary ductoscopy, Duct-washing cytology, Ductal carcinoma in situ, Breast cancer

## Abstract

**Objective:**

Conventional cytological diagnosis including duct-washing cytology (DWC) is sometimes performed using ductal epithelial cells collected during mammary ductoscopy; it is useful for detection of early-stage breast cancer such as ductal carcinoma in situ (DCIS). However, conventional cytological diagnosis focuses exclusively on cellular morphology; false negatives and false positives may be caused by inadequate specimen preparation (triggering cell degeneration) or poor examiner diagnostic skills. Molecular diagnosis using RNA biomarkers is expected to compensate for the weaknesses of cytological diagnosis. We previously employed microarray analysis to identify highly expressed genes in DCIS, suggesting that they may be useful for DCIS diagnosis. Here, we explored whether DWC samples yielded RNA of sufficient quantity and quality for RNA biomarker-based diagnosis.

**Results:**

We extracted RNAs from 37 DWC samples. RNA from 12 samples exhibited RNA integrities of  ≥ 6, indicative of moderate-to-high quality. We then showed that cocaine and amphetamine regulated transcript prepropeptide (CARTPT) and breast cancer-associated transcript 54 (BRCAT54) mRNA—previously shown by microarray analysis to be highly expressed in DCIS—were detectable in these samples. Therefore, DWC samples may be useful for molecular diagnosis involving RNA biomarkers.

**Supplementary Information:**

The online version contains supplementary material available at 10.1186/s13104-022-05928-1.

## Introduction

Breast cancer (BC) originates in the mammary ducts. Some cancers may be metastatic, thus progressing from non-invasive to invasive. Because of substantial advances in breast imaging tools, ductal carcinoma in situ (DCIS) constitutes approximately 20% of newly diagnosed BCs; its proportion is increasing [1, 2]. Early BC detection improves prognosis. Abnormal nipple discharge (ND) may be the earliest presenting symptom of BC; substances in such discharge may yield detailed information regarding BC. Indeed, carcinoembryonic antigen in ND has served as a BC biomarker [3, 4]. At present, ND is only regarded as an aid to diagnosis; cytological and/or histological evaluation is considered essential for accurate diagnosis. Preoperative cytological diagnosis can be accurate if atypical cells are present in small samples. Ultrasound-guided fine needle aspiration biopsy is widely used to collect material from the ventral surfaces of mammary glands [5]. However, conventional cytological diagnosis focuses exclusively on cellular morphology; false negatives and false positives may be caused by inadequate specimen preparation (triggering cell degeneration) or poor examiner diagnostic skills.

Mammary ductoscopy (MD) allows direct visualisation of the ductal lumen using a fibreoptic microendoscope, as well as the collection of cytological specimens for duct-washing cytology (DWC); this is an alternative form of preoperative BC diagnosis. It would be clinically useful if surplus DWC specimens could be used for RNA extraction and subsequent molecular diagnosis involving RNA biomarkers. Here, we explored whether RNA of sufficient quantity and quality could be recovered from DWC specimens of DCIS patients. Furthermore, we explored whether the RNA contained cocaine- and amphetamine-regulated transcript prepropeptide (CARTPT) and breast cancer-associated transcript 54 (BRCAT54) mRNAs, which a previous microarray study found to be highly expressed in DCIS [6].

## Main text

### Materials and methods

#### Surplus specimens of duct-washing cytology

The surplus tissues of 45 specimens collected for DWC at Musashi Kosugi Hospital from December 2017 to October 2019 were used. All patients presented with ND. Observation and specimen sampling were carried out as follows. First, nipple duct dilatation was performed using bougies; an 18-G Surflo intravenous catheter was then inserted. The duct was washed with a lidocaine solution, and the solution was collected. Second, Solid Fibrescope MS-611 (FiberTech Co. Ltd. Chiba, Japan) was inserted and the lesion was directly observed. MD allows direct observation of the ductal interior; a fibre of outer diameter 0.8 mm is placed within an 18-G Surflaw needle. Finally, aspiration biopsy was performed using an 18-G Surflo intravenous catheter after removal of the fibrescope [7, 8].

#### Extract RNA from duct-washing cytology specimens

Total RNA was extracted from specimens using RNAiso-Plus (Takara-Bio, Kusatsu, Japan), generally in accordance with the manufacturer’s instructions; however, all steps were performed below 4 °C and linear acrylamide (Thermo Fisher Scientific, Waltham, MA, USA) was added as a co-precipitant at the step of isopropyl alcohol precipitation (Additional file 1: Fig. S1). Each RNA sample was resuspended in 10 μL RNase/DNase-free distilled water.

#### Assessment of the quantity and quality of DWC specimens

RNA concentrations were determined using a QuantiFluor RNA System (Promega, Madison, WI, USA). The A260/A280 ratios of RNAs extracted from DWC specimens were determined using a NanoDrop 1000 spectrophotometer (Thermo Fisher Scientific).

#### RNA quality assessment

The extracted RNA was further analyzed using the Agilent 2100 Bioanalyzer (Agilent, Santa Clara, USA) in combination with the RNA 6000 Nano Lab Chip kit (Agilent). The quality of RNA was assessed by RNA integrity number (RIN) [9].

#### Real-time reverse-transcription PCR

The levels of mRNAs encoding CARTPT, BRCAT54, and hypoxanthine phosphoribosyltransferase 1 (HPRT-1) (internal standard) were measured via quantitative reverse-transcription (qRT)-PCR using the SYBR Green Assay (Thermo Fisher). PCR products were monitored on a DICE Real-Time PCR System (Takara-Bio).

The primer sequences (all 5ʹ–3ʹ) were: CARTPT forward, AGAAGGAGCTGACGAAGCG; CARTPT reverse, ACACAGCTTCCCGATCCTTG; BRCAT54 forward, CCTGCAGGAAAATGCAGTGAAG; BRCAT54 reverse CACCATCACATTGCTGACTTCCA; and HPRT1 forward, CATTATGCTGAGGATTTGGAAAGG; HPRT1 reverse CTTGAGCACACAGAGGGCTACA. Our qRT-PCR analyses to evaluate CARTPT, BRCAT54, and HPRT-1 mRNA levels were performed in triplicate.

#### Immunobiological staining

Of the 37 patients who underwent MD, eight underwent surgery and seven turned out to have malignant tumors. For immunopathological diagnosis, they underwent immunobiological staining for estrogen receptor (ER), progesterone receptor (PR), and human epidermal growth factor receptor 2 (HER2) (Additional file 1: Table S1).

#### Statistical analysis

Spearman’s rank correlation analyses between RNA yields and RIN were performed using EZR, a modified version of R software (version 1.53; R Foundation for Statistical Computing, Vienna, Austria) with additional biostatistical functions [10].

## Results and discussion

**Fig. 1:**
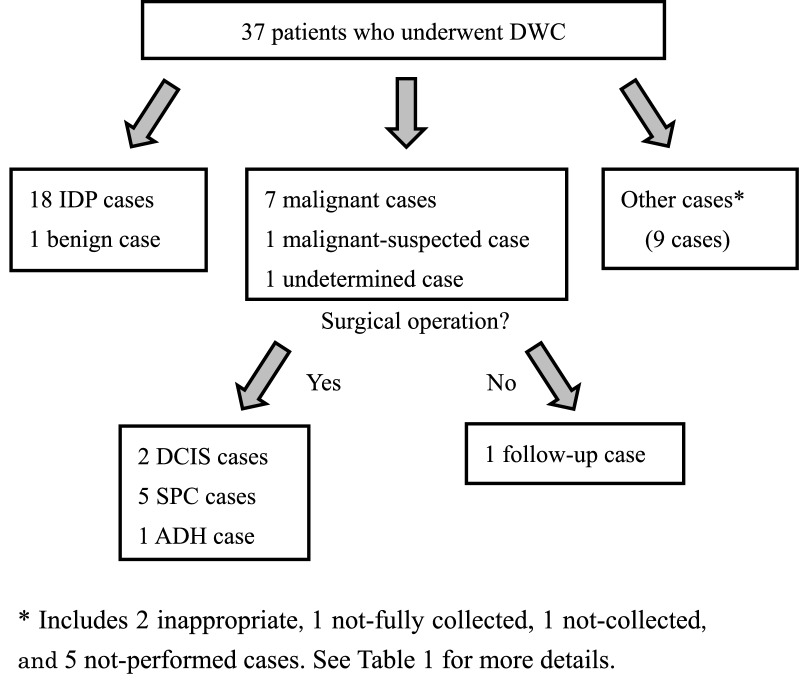
Flow chart of patient selection. *DWC* duct-washing cytology; *IDP* intraductal papilloma; *DCIS* ductal carcinoma in situ; *SPC* solid papillary carcinoma; *ADH* atypical ductal hyperplasia

Figure 1 shows a flowchart of the selection of 37 patients. We obtained DWC specimens by MD and extracted RNA. Of the 37 patients, eight had malignancies or suspected malignancies; one patient’s results were indeterminate. Of nine patients (eight with malignancies and one with indeterminate findings), two were diagnosed with DCIS, five were diagnosed with solid papillary carcinomas (SPCs), one was diagnosed with atypical ductal hyperplasia (ADH), and one had indeterminate findings; all underwent surgery, except the patient with indeterminate findings. One patient with suspected malignancy did not undergo surgery because she was under observation at another hospital. SPC is a particular subtype of DCIS that exhibits a full papillary growth pattern; it is described as a separate category of “intraductal papillary lesion” in the 4th edition of the World Health Organisation criteria [11]. ADH is a pre-cancerous state: a lesion that has both benign and malignant features. Intraductal papilloma (IDP), wart-like lump that develops in one or more mammary ducts, is regarded as a benign condition.

Patient information including ages, DWC diagnoses, MD findings, and the RINs and amounts of isolated RNAs are shown in Table 1. Of the nine patients mentioned above, eight underwent surgery, and seven had malignancies (Additional file 1: Table S1). Immunobiological staining revealed that all seven were ER- and PR-positive with positive cell proportions of at least 80%; six malignancies were HER2-negative. The invasion grades were very low (0 or 0.3); the spread ranged from 2.0 to 10.5 cm (mean, 5.3 cm).

**Table 1:** The clinical information of the patients

Patients’ no.	Sample no.	y/o	DWC	MD finding	RNA yields (ng)	RIN
1	1	42	BC	BC	1448.5	6.2
2	2	71	BC	IDP	803.7	7.9
	3				1030.5	8.6
3	4	37	BC	BC-suspected	487.2	3.4
	5				488.9	7.2
4	6	68	BC-suspected	BC-suspected	52.2	8.2
	7				57.6	7.6
5	8	31	BC-suspected	BC	176.7	6.7
6	9	37	BC	BC	97.9	nd
7	10	41	Undetermined	BC-suspected	125.9	7.4
8	11	41	BC-suspected	BC	97.6	4.2
	12				289.8	3.5
9	13	49	BC^a^	IDP	123.2	3.7
	14				205.8	3.3
10	15	62	IDP	IDP	655.6	8.1
11	16	40	IDP	IDP	277.7	7.6
	17				252.8	5.8
12	18	53	IDP	IDP	54.7	7.2
13	19	66	IDP	IDP	121.5	7.1
14	20	43	IDP	IDP-suspected	2419.4	2.4
15	21	59	IDP	IDP	353.8	2.4
16	22	45	IDP	IDP	174.3	2.8
17	23	47	IDP	IDP	63.5	2.9
18	24	67	IDP	IDP	79.4	1.0
19	25	49	IDP	IDP	141.9	4.2
	26				135.4	3.7
20	27	37	IDP	IDP	2673.0	1.0
21	28	40	IDP	IDP	97.8	5.2
22	29	41	IDP	IDP	78.6	3.2
23	30	49	IDP	IDP	51.9	5.5
24	31	43	IDP	BC	546.3	3.5
25	32	38	IDP	BC	97.2	2.1
26	33	44	IDP	BC	153.1	5.1
27	34	36	IDP	BC	183.5	2.5
28	35	62	Inappropriate	IDP	237.9	4.5
29	36	33	Inappropriate	IDP	198.1	3.7
30	37	45	Benign^b^	IDP	89.7	1.0
31	38	41	Not performed^c^	Duct ectasia	1371.7	2.0
32	39	47	Not performed^c^	Duct ectasia	149.9	3.2
33	40	40	Not performed^c^	Duct ectasia	255.1	5.5
34	41	49	Not performed^c^	Duct ectasia	331.7	4.0
35	42	41	Not performed^c^	Duct ectasia	732.9	2.6
	43				530.2	2.6
36	44	57	Not fully collected^d^	(Ruptured the duct)	75.4	2.8
37	45	67	Not collected	Mastopathy-suspected	1072.8	3.4

Based on DWC, 8 patients were diagnosed as BC (patient no. 1–3, 6, and 9) and BC suspected (patient no. 4, 5, and 8), and one patient (patient no. 7) was undetermined

*nd* not determined

^a^Under observation at another hospital

^b^Hyperplastic epithelium and stroma

^c^Not performed due to the absence of elevated lesions

^d^Ruptured the ducts and air leaked out

RNAs of moderate-to-high quality (RIN  ≥ 6) were isolated from 12 samples (1–3, 5–8, 10, 15, 16, 18, and 19) (Table 1). Spearman’s rank correlation analysis for 44 samples (Sample no. 9 was excluded because its RIN was not determined) revealed that there was no correlation between the amounts and RINs of RNA isolated from the specimens (r  = − 0.08).

Of these, eight were malignant (six SPC and two DCIS); four were benign (four IDP). During the follow-up period, the tumours of two patients became malignant. Patient no. 16 was recently diagnosed with BC by puncture of a peripheral mass. Patient no. 27 eventually underwent surgery and diagnosed as mucinous carcinoma located in the peripheral region of the duct. We selected five samples (three malignant and two benign) with high RNA yields (Table 2) for quantitative RT-PCR analysis of CARTPT and BRCAT54. These mRNAs were chosen because microarray analysis previously showed that they were highly upregulated in DCIS- and SPC-derived tissues [12, 13] In addition, the levels of mRNA encoding HPRT-1 (a representative housekeeping gene) were analysed. RNA from the breast tumour tissue of an SPC patient—previously used in array analysis [6]—served as the positive control (sample no. 0). CARTPT and HPRT-1 mRNAs were detected in all samples. BRCAT54 mRNA was detected in the samples although expressed at a quite low level in one IDP-derived sample (No.16) (Table 2; Additional file 1: Fig. S2). Dissociation curves with single peaks were obtained for the samples (Additional file 1: Fig. S2), indicating that non-specific PCR amplicons were not generated.

**Table 2:** Result of qRT-PCR analysis

Patients’ no.	Sample no.	Pathological diagnosis	HPRT-1 (Ct value)	CARTPT/HPRT-1 (RQ)	BRCAT54/HPRT-1 (RQ)
0^a^	0	SPC	24.0	1.0	1.0
1	1	SPC	27.2	2.8	1.4
2	2	SPC	26.2	0.1	5.1
2	3	SPC	25.9	0.1	5.4
10	15	IDP	27.1	14.2	2.0
11	16	IDP	27.9	0.2	0.0

^a^RNA from the FFPE sample of an SPC patient, in which BRCAT54, CARTPT and HPRT-1 were previously shown to be highly expressed by array analysis, was also analyzed as a positive control

To the best of our knowledge, no report about RNA extraction from MD samples has been published so far, although there are a few reports describing the analysis of DNA methylation in MD samples [14, 15]. This study has clinical relevance in that we demonstrated a possibility that RNA of acceptable quality and quantity could be extracted from DWC specimens and applied to RNA-based pre-operative diagnosis of breast carcinoma.

In summary, we used MD to examine mass lesions in the mammary ducts and extracted RNA from DWC samples; many extracts exhibited moderate-to-high RNA quality. We found that mRNAs abundantly expressed in SPC or IDP can be detected via qRT-PCR.

## Limitation

There are some limitations in this study. For example, the sample size of this study is small, which conceivably weakens the results deduced from the provided data. On a relevant note, the clinical usability of BRCAT54 and CARTPT mRNAs as biomarkers has not been evaluated. Further, our method is currently applicable only for patients who exhibit mass lesions in their mammary ducts and may be difficult to apply for patients with small intraductal lesions, because only a few cells can be conceivably recovered from such small lesions.

## Supplementary Information

**Additional file 1: Figure S1.** Flowchart of RNA extraction. **Figure S2.** Detection of BRCAT54-, CARTPT- and HPRT-1-encoding mRNAs in RNA isolated from representative DWC samples. **Table S1.** Pathological findings.

## Data Availability

Not applicable.

## Abbreviations

DCIS: Ductal carcinoma in situ
DWC: Duct-washing cytology
MD: Mammary ductoscopy
BC: Breast cancer
RIN: RNA integrity number
SPC: Solid papillary carcinoma
CARTPT: Cocaine and amphetamine regulated transcript prepropeptide
BRCAT54: Breast cancer-associated transcript 54
HPRT-1: Hypoxanthine phosphoribosyltransferase 1
ADH: Atypical ductal hyperplasia
IDP: Intraductal papilloma
ER: Estrogen receptor
PR: Progesterone receptor
HER2: Human epidermal growth factor receptor 2

## References

[1] Si W, Li Y, Han Y (2015). Epidemiological and clinicopathological trends of breast cancer in Chinese patients during 1993 to 2013: a retrospective study. Medicine.

[2] Adamovich TL, Simmons RM (2003). Ductal carcinoma in situ with microinvasion. Am J Surg.

[3] Mori T, Inaji H, Koyama H (1992). Evaluation of an improved dot-immunobinding assay for Carcinoembryonic Antigen determination in nipple discharge in early breast cancer: results of a multicenter study. Jpn J Clin Oncol.

[4] Oda M, Makita M, Iwaya K (2012). High levels of DJ-1 protein in nipple fluid of patients with breast cancer. Cancer Sci.

[5] Yamaguchi R, Tsuchiya SΙ, Koshikawa T (2012). Diagnostic accuracy of fine-needle aspiration cytology of the breast in Japan: report from the Working Group on the Accuracy of Breast Fine-Needle Aspiration Cytology of the Japanese Society of Clinical Cytology. Oncol Rep.

[6] Jikuzono T, Manabe E, Kure S (2021). Microarray analysis of Ductal carcinoma in situ samples obtained by puncture from a surgical resection specimen. BMC Res Notes.

[7] Makita M, Akiyama F, Gomi N (2006). Endoscopic and histologic findings of intraductal lesions presenting with nipple discharge. Breast J.

[8] Makita M, Sakamoto G, Akiyama F (1991). Duct endoscopy and endoscopic biopsy in the evaluation of nipple discharge. Breast Cancer Res Treat.

[9] Schroeder A, Mueller O, Stocker S (2006). The RIN: an RNA integrity number for assigning integrity values to RNA measurements. BMC Mol Biol.

[10] Kanda Y (2013). Investigation of the freely available easy-to-use software ‘EZR’ for medical statistics. Bone Marrow Transpl.

[11] Lakhani SR, Ellis IO, Schnitt SJ (2012). WHO classification of tumours of the breast.

[12] Lu K, Wang X, Zhang W (2020). Clinicopathological and genomic features of breast mucinous carcinoma. Breast.

[13] Yang W, Qian Y, Gao K (2021). LncRNA BRCAT54 inhibits the tumorigenesis of non-small cell lung cancer by binding to RPS9 to transcriptionally regulate JAK-STAT and calcium pathway genes. Carcinogenesis.

[14] Zhu W, Qin W, Zhang K (2012). Trans-resveratrol alters mammary promoter hypermethylation in women at increased risk for breast cancer. Nutr Cancer.

[15] Fackler MJ, Rivers A, Teo WW (2009). Hypermethylated genes as biomarkers of cancer in women with pathologic nipple discharge. Clin Cancer Res.

