# Supplementary material for: RNA recovery from specimens of duct-washing cytology performed contemporaneously with mammary ductoscopy
Source: BMC Res Notes. 2022 Feb 10;15:34. doi: 10.1186/s13104-022-05928-1 (PMC8830173; doi:10.1186/s13104-022-05928-1)
Supplement: Supplementary file 1 — Additional file 1: Figure S1. Flowchart of RNA extraction. Figure S2. Detection of BRCAT54-, CARTPT- and HPRT-1-encoding mRNAs in RNA isolated from representative DWC samples. Table S1. Pathological findings. [file 13104_2022_5928_MOESM1_ESM.docx]

Add DWC sample to 1ml of RNAiso Plus and keep frozen

incubate on ice for 5 minutes

After thawing, add 200 μl of chloroform and shake for 15 seconds

centrifuge for 10 minutes at 13,500 rpm at 4°C

Transfer the upper water layer to a new centrifuge tube

Add 500 μl of isopropanol

centrifuge for 10 minutes at 13,500 rpm at 4℃

at 4°C leave the tube on ice for 10 minutes

leave the tube on ice for 10 minutes

Discard the supernatant, leaving the sediment

Add 1 ml of cold 70% ethanol

centrifuge for 5 minutes at 13,500 rpm at 4℃

at 4°C leave the tube on ice for 10 minutes

Discard the supernatant, leaving the sediment

Add 500 μl of cold 70% ethanol

centrifuge for 5 minutes at 13,500 rpm at 4°C

Discard the supernatant, leaving the sediment

air dry

Dissolve with 10 μl of water

Fig. S1 Procedure of RNA extraction.

BRCAT54


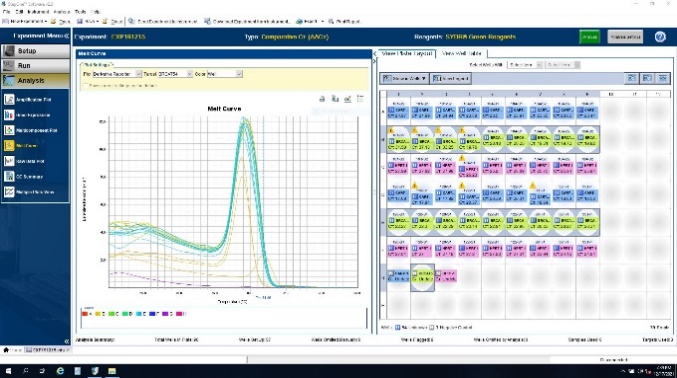

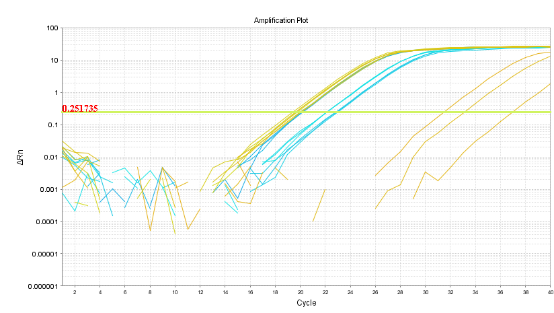


CARTPT


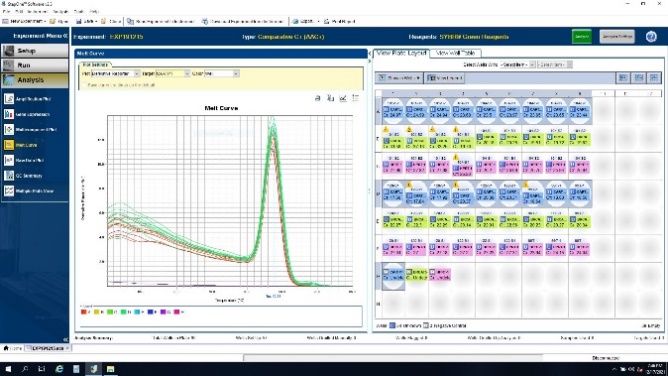

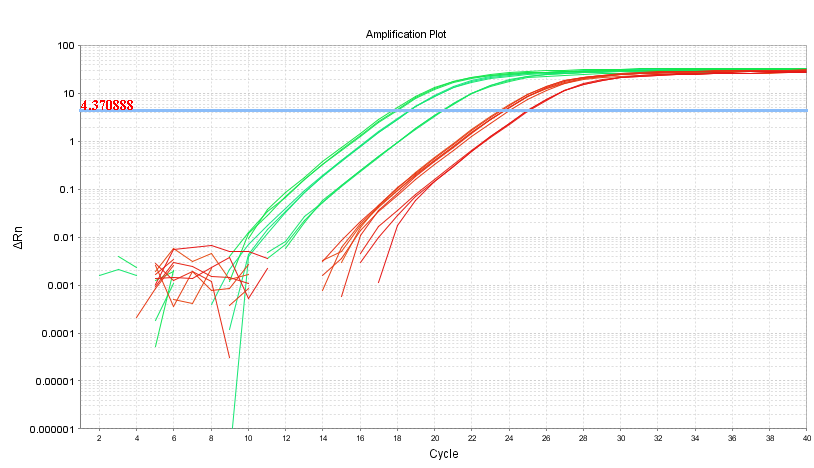


HPRT-1


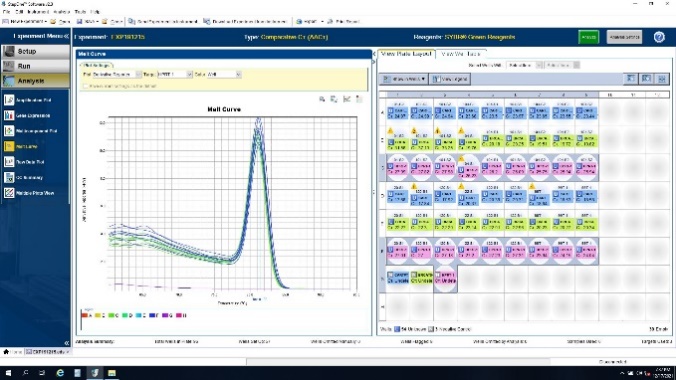

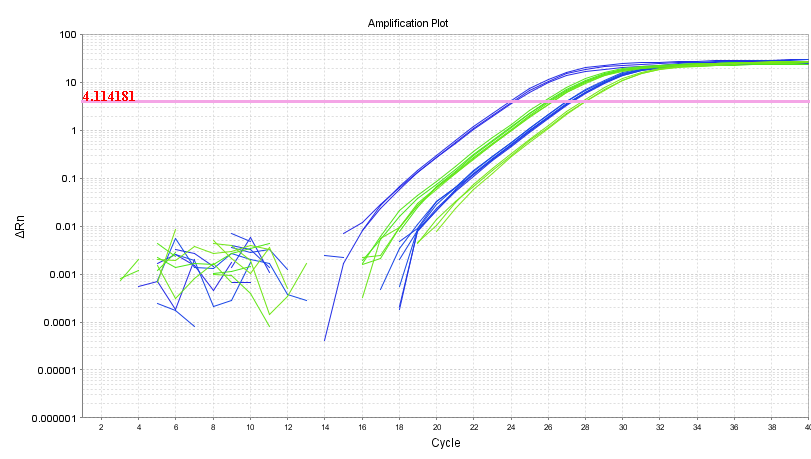


Fig. S2 Detection of BRCAT54, CARTPT and HPRT-1 mRNAs in RNA isolated from representative DWC samples. The amplification plots and dissociation curves (insets) of these mRNAs are shown.

Table S1 Pathological findings

| Patients' No. | Pathological diagnosis | ER-positive rate | PgR-positive rate | HER2 | invasion size [cm] | spreading size [cm] |
| --- | --- | --- | --- | --- | --- | --- |
| 1 | SPC | 90％ | 90％ | 0 | 0 | 7.0 |
| 2 | SPC | 90％ | 90％ | 0 | 0 | 4.5 |
| 3 | SPC | 90％ | 90％ | 0 | 0.3 | 10.5 |
| 4 | DCIS | 90％ | 80％ | 0 | 0 | 2.0 |
| 5 | SPC | 90％ | 90％ | 0 | 0 | 4.7 |
| 6 | DCIS | 90％ | 90％ | 2＋ | 0 | 8.1 |
| 7 | SPC | 80％ | 90％ | 0 | 0 | 5.7 |
| 8 | ADH |  |  |  |  |  |
